# Network Analysis to Identify Multi-Omic Correlations in the Lower Airways of Children With Cystic Fibrosis

**DOI:** 10.3389/fcimb.2022.805170

**Published:** 2022-03-10

**Authors:** John B. O’Connor, Madison Mottlowitz, Monica E. Kruk, Alan Mickelson, Brandie D. Wagner, Jonathan Kirk Harris, Christine H. Wendt, Theresa A. Laguna

**Affiliations:** ^1^ Department of Pediatrics, Division of Pulmonary and Sleep Medicine, Ann & Robert H. Lurie Children’s Hospital of Chicago, Chicago, IL, United States; ^2^ Department of Biochemistry, Molecular Biology and Biophysics, University of Minnesota, Minneapolis, MN, United States; ^3^ Department of Medicine, University of Minnesota, Minneapolis VA Medical Center, Minneapolis, MN, United States; ^4^ School of Medicine, University of Colorado, Aurora, CO, United States; ^5^ Colorado School of Public Health, University of Colorado Denver, Aurora, CO, United States; ^6^ Northwestern University Feinberg School of Medicine, Chicago, IL, United States

**Keywords:** cystic fibrosis, metabolomics, microbiota (16S), pediatrics, bronchoalevolar lavage, infection, inflammation

## Abstract

The leading cause of morbidity and mortality in cystic fibrosis (CF) is progressive lung disease secondary to chronic airway infection and inflammation; however, what drives CF airway infection and inflammation is not well understood. By providing a physiological snapshot of the airway, metabolomics can provide insight into these processes. Linking metabolomic data with microbiome data and phenotypic measures can reveal complex relationships between metabolites, lower airway bacterial communities, and disease outcomes. In this study, we characterize the airway metabolome in bronchoalveolar lavage fluid (BALF) samples from persons with CF (PWCF) and disease control (DC) subjects and use multi-omic network analysis to identify correlations with the airway microbiome. The Biocrates targeted liquid chromatography mass spectrometry (LC-MS) platform was used to measure 409 metabolomic features in BALF obtained during clinically indicated bronchoscopy. Total bacterial load (TBL) was measured using quantitative polymerase chain reaction (qPCR). The Qiagen EZ1 Advanced automated extraction platform was used to extract DNA, and bacterial profiling was performed using 16S sequencing. Differences in metabolomic features across disease groups were assessed univariately using Wilcoxon rank sum tests, and Random forest (RF) was used to identify features that discriminated across the groups. Features were compared to TBL and markers of inflammation, including white blood cell count (WBC) and percent neutrophils. Sparse supervised canonical correlation network analysis (SsCCNet) was used to assess multi-omic correlations. The CF metabolome was characterized by increased amino acids and decreased acylcarnitines. Amino acids and acylcarnitines were also among the features most strongly correlated with inflammation and bacterial burden. RF identified strong metabolomic predictors of CF status, including L-methionine-S-oxide. SsCCNet identified correlations between the metabolome and the microbiome, including correlations between a traditional CF pathogen, *Staphylococcus*, a group of nontraditional taxa, including *Prevotella*, and a subnetwork of specific metabolomic markers. In conclusion, our work identified metabolomic characteristics unique to the CF airway and uncovered multi-omic correlations that merit additional study.

## Introduction

Progressive obstructive lung disease is the leading cause of morbidity and mortality in persons with cystic fibrosis (PWCF) (Gibson et al., 2003; Rowe et al., 2005); however, the complex interplay between bacterial communities infecting the lower airway, inflammation, and lung destruction remains to be defined. Despite the development of exciting new therapies that have been shown to improve clinical outcomes (Rowe et al., 2014; Bernarde et al., 2015; Hisert et al., 2017), airway infection and inflammation are persistent in PWCF. While chronic infection is a hallmark of CF lung disease (Govan and Nelson, 1992; Sagel and Accurso, 2002; Gibson et al., 2003; Nichols et al., 2008), the inflammatory response is persistent and heightened relative to bacterial burden and has been observed in the CF airway even in the absence of infection in both human and animal models (Hendry et al., 1999; Sagel and Accurso, 2002; Chmiel and Davis, 2003; ChmiEL and Konstan, 2010; Keiser et al., 2015; O’Connor et al., 2021). Improving our understanding of the pathophysiological processes associated with airway infection and inflammation can provide insight into the mechanisms of CF lung disease and identify therapeutic targets.

Detecting, identifying, and treating infections in the lower airway are cornerstones of clinical management to preserve lung function in CF; however, surveillance cultures of airway secretions alone have limitations based on sample type and scope. While molecular-based approaches like 16S ribosomal RNA (rRNA) sequencing have identified communities of bacteria present in the upper and lower airways of PWCF that may diversify and shift over time (Coenye et al., 2002; Rogers et al., 2004; Sibley et al., 2006; Bittar et al., 2008; Willner et al., 2012; Lim et al., 2014; Hoen et al., 2015; LiPuma, 2015; Tracy et al., 2015; Prevaes et al., 2016; Feigelman et al., 2017; Jorth et al., 2019), this method in isolation does not provide insight into active community metabolism and dynamics of an ever-shifting airway environment (Quinn et al., 2016). Metabolomics, which profiles endogenous and microbial metabolites within biological specimens, can provide a comprehensive physiological snapshot of metabolic activity in the lower airway environment, help identify putative biomarkers of disease, and shed light on the mechanisms of infection and disease pathogenesis (Serkova et al., 2011). Combining the power of mass-spectrometry (MS) based metabolomic approaches with 16S rRNA sequencing data can provide unique and valuable insight into the mechanisms driving infection and inflammation in the CF airway (Quinn et al., 2016).

While metabolomics has been performed on samples from PWCF, including sputum (Jones et al., 2000; Palmer et al., 2007; Bensel et al., 2011; Yang et al., 2012; Twomey et al., 2013; Quinn et al., 2016) and exhaled breath (Barker et al., 2006; Celio et al., 2006; Newport et al., 2009; Robroeks et al., 2010; Montuschi et al., 2012; Monge et al., 2013), studies in bronchoalveolar lavage fluid (BALF) have been limited. Previous work characterizing the BALF metabolome has identified metabolites associated with inflammation, bronchiectasis, and structural lung disease (Wolak et al., 2009; Esther et al., 2015; Esther et al., 2016); however, those studies were limited in sample number and did not include non-CF DC subjects, which can provide valuable comparisons to identify CF-specific metabolomic signatures (Masood et al., 2021). Furthermore, those studies did not compare metabolomic features with the lower airway microbiome. Modern multi-omics approaches that link metabolomic and microbiome genomic data have the power to reveal complex relationships between metabolites, lower airway bacterial communities and phenotypic measures (Chen et al., 2013; Quinn et al., 2016; Shi et al., 2019). Previous work with the microbiome and metabolome in CF sputum has highlighted complex relationships between bacterial communities, metabolomic characteristics, and clinical features (Twomey et al., 2013; Quinn et al., 2016; Quinn et al., 2019; Hahn et al., 2020; Raghuvanshi et al., 2020). Increased abundance of CF pathogens has been found to correlate with metabolomic disease states characterized by increased peptides and amino acids (Quinn et al., 2019) and with increased markers of inflammation (Zemanick et al., 2015). Additionally, strong correlations have been identified between the presence of strict anaerobes in sputum and the abundance of putrescine, pyruvate, and lactate (Twomey et al., 2013). While we would expect to see similar correlations between microbes and metabolomic features in the lower CF airway, no multi-omics correlations have been reported in bronchoalveolar lavage fluid (BALF) to date.

Through the integration of targeted metabolomics, microbiome data, and phenotypic measures, including cellular markers of inflammation, our objective was to apply a modern multi-omics network analysis approach to investigate the complex relationships between the airway microbiome and metabolomic features in BALF from PWCF across the age spectrum.

## Materials And Methods

### Study Design and Population

Stored BALF previously collected from PWCF and DCs undergoing clinically indicated bronchoscopies under institutional IRB-approved protocols at multiple sites throughout the United States was used. BALF from PWCF was collected at 13 CF centers as part of a previous multi-center study (Zemanick et al., 2017). BALF from DC subjects, defined as those without a confirmed diagnosis of CF, was collected at Children’s Hospital of Colorado and the University of Minnesota. Associated demographic and clinical data, including comorbidities, lung function measures, cell counts, and culture data, were collected through the electronic medical record and the Cystic Fibrosis Foundation Patient Registry (CFFPR). Informed consent, adolescent assent, and parental permission along with HIPPA (Health Insurance Portability and Accountability Act of 1996) authorization from subjects and/or legal guardians was obtained according to each individual site’s IRB rules and regulations.

### Sample Collection and Processing

Flexible bronchoscopy with lavage was performed on subjects in accordance with each site’s standard of care guidelines and leftover BALF was collected and stored for research. Neat unprocessed samples were directly aliquoted and set aside, and volume permitting, remaining sample was centrifuged at 250 x G for 10 minutes at 4°C followed by separation of supernatant and pellet. Then, following that separation, the supernatant was centrifuged again at 4000 x G for 20 minutes at 4°C and separated again. Finally, all neat, pellet, and supernatant samples were aliquoted and stored at -80°C. All samples were shipped to Lurie Children’s Hospital of Chicago, the central site for sample storage, on dry ice. Similarly, samples were shipped on dry ice to collaborating sites, which included Children’s Hospital Colorado, where quantitative polymerase chain reaction (qPCR) and 16S rRNA sequencing were performed, and the University of Minnesota, where metabolomic profiling was conducted.

### Metabolomic Profiling

Supernatant samples were vortexed and centrifuged again at 5000 x G for 5 minutes at 4°C followed by separation of the pellet and supernatant for the removal of additional debris. 200 μL of supernatant was manually loaded onto the Biocrates Life Sciences Absolute IDQ p400 HR (Biocrates Life Sciences catalog number 21018) deep 96-well polypropylene plate. Samples were pipetted in four 50 µL increments. The addition of each increment was followed by drying under liquid nitrogen for 30 minutes. The supernatant was pipetted into the designated well in a randomized plate layout created in MetIDQ and the plate was sealed with a clean silicon mat. A Thermo Scientific, Q Exactive TM, Hybrid Quadrupole-Orbitrap TM, mass spectrometer equipped with a Thermo Scientific Ultimate 3000 UHPLC and an autosampler was used for metabolomic characterization analysis. The autosampler was set to collect eluent from 0.2 to 1.5-minute retention times. The Xcalibur Qual Browser software was used for MS data processing. Metabolomic feature concentrations were quantified using the integrated MetIDQ Biocrates software (Wenk, 2005). The Biocrates platform was used to measure a total of 409 metabolomic features set by the manufacturer using isotope-labeled calibration standards from 8 different families (number of features), including Acylcarnitines (55), Amino Acids (21), Biogenic amines (21), Cholesterol Esters (14), Glycerides (60), Glycerophospholipids (197), Sphingolipids (40), and Sugars (1). Features included groups of isomers that could not be separated by chromatography. In addition to BALF samples and calibration standards, on each plate, 3 zero sample replicates were used for background noise calculation, 1 blank sample was used for background subtraction, and 3 plasma quality control samples spiked with different amounts of isotope-labeled internal standard were used to assess variation between plates. The mid-level plasma quality control sample was pipetted after every 20 wells of the plate to assess variation between plate well locations. The limit of detection (LOD) for each metabolomic feature was calculated using the MetIDQ software and was equal to 3 times the median background noise approximation.

### Microbiome Analysis

Detailed information regarding the microbiome analysis is included in the supplementary material. Briefly, the Qiagen EZ1 advanced automated extraction platform (Qiagen, Valencia, CA, USA) was used to extract DNA from the samples in accordance with the manufacturer’s instruction. Total bacterial load (TBL) was measured using a quantitative polymerase chain reaction (qPCR) assay as previously done on CF BALF samples (Nadkarni et al., 2002; Zemanick et al., 2010; O’Connor et al., 2021). Broad range amplification and sequence analysis of the V1/V2 variable region (27F/338R) of the 16S rRNA gene was used to profile airway bacterial taxa in a process previously reported (Hara et al., 2012; Markle et al., 2013; Laguna et al., 2016; Zemanick et al., 2017; O’Connor et al., 2021). All unique sequences were assigned taxonomic information using SINA (Pruesse et al., 2012). Operational taxonomic units (OTUs) were generated by totaling counts for sequences assigned to the same taxonomic group.

### Data Processing and Statistical Analysis

For the analysis of demographic data, categorical variables were compared across disease groups using Chi-squared tests and Fisher’s exact tests, and comparison of numerical variables across groups was performed using Wilcoxon rank-based tests. For metabolomic analysis, preprocessing was performed using MetaboAnalystR (v3.0.3). Features with 50% of the values outside the limit of detection and features with constant values across the sample set were excluded from analysis. Missing values were imputed using K-nearest neighbors (KNN) imputation, which has been previously described (Troyanskaya et al., 2001). Metabolomic feature concentrations were scaled and centered for normalization prior to all analyses. Differences in feature concentrations across disease groups were assessed univariately using Wilcoxon rank sum tests, p-values were corrected using false discovery rate (Benjamini and Hochberg, 1995), and fold change was calculated as the ratio between group means using data before column normalization. Random forests consisting of 5,000 classification trees were used to identify the subset of top metabolomic features which best discriminated across the groups (Breiman, 2001). Relationships between metabolomic features and inflammatory cell markers and TBL were assessed using Spearman correlations. A canonical correlation-based approach was used to evaluate associations between metabolomics and 16S rRNA gene sequencing data (SsCCNet function in R) for the subset of samples with both data types. Phenotypes of interest included CF status, which was determined based on whether the subject had a confirmed diagnosis of CF, as well as markers of inflammation, which included white blood cell count and percent neutrophils. Sparse supervised canonical correlation network analysis (SsCCNet) on all the samples incorporated the CF phenotype. Network analysis was also run on the CF only sample set both unsupervised (no phenotypic outcome) and incorporating inflammation as the phenotypic variable (Shi et al., 2019). Statistical analyses were performed with R version 3.5.1 (R Foundation for Statistical Computing, Vienna Austria).

## Results

### Study Population

Ninety BALF samples were collected, which included 68 (76%) from PWCF and 22 (24%) from DCs. Demographics and relevant clinical characteristics are presented in **Table 1**. PWCF had more airway inflammation and more positive BALF cultures (p=0.01), with higher rates of *Pseudomonas aeruginosa* (p=0.02) and methicillin-susceptible *Staphylococcus aureus* (p=0.02). PWCF were over three times as likely to be treated with antibiotics at the time of sample collection (p<0.01). Indications and primary diagnoses of DC samples are summarized in **Supplementary Table 1**.

**Table 1:** Data are presented as n, median (range) or n (%), unless otherwise stated.

	CF (n = 68)	Disease Control (n = 22)	P-value
Age years, median (range)	12 (0.5-28.0)	7.2 (1.3-19.0)	0.07
<2 years, number (%)	4 (6%)	3 (14%)	0.21*
2-5 years, number (%)	9 (13%)	5 (23%)	
6-10 years, number (%)	16 (24%)	7 (32%)	
11-17 years, number (%)	26 (38%)	6 (27%)	
18 years and older, number (%)	13 (19%)	1 (5%)	
Female, number (%)	32 (47%)	12 (55%)	0.54
Weight (kg), median (range) (data available)	43.0 (6.4-87.0) (N=64)	27.2 (9.9-83.0) (N=22)	0.18
Height (cm), median (range) (data available)	149.7 (64.5-185.4) (N=64)	125.7 (74.0-181.9) (N=22)	0.12
Genotype, data available	N=59	N/A	:_
F508del/F508del, number (%)	37 (63%)	N/A	:_
F508del/other, number (%)	17 (29%)	N/A	:_
Other/other, number (%)	5 (8%)	N/A	:_
FEV1 % predicted, median (range) (data available)	80.5 (41.0-125.0) (N=50)	87.5 (38.0-121.0) (N=12)	0.19
BALF Cell Counts, data available	N=68	N=22	
White blood cells, median (range) (data available)	620.0 (0.0-41167.0) (N=68)	231.0 (38.0-2555.0) (N=22)	0.34
Percent Neutrophils, median (range) (data available)	67.0 (0-100.0) (N=57)	4.5 (1.0-100.0) (N=22)	<0.01
Percent Lymphocytes, median (range) (data available)	3.0 (0-28.0) (N=55)	10.0 (0-65.0) (N=22)	0.02
BALF culture results, data available	N=64	N=19	
Negative, number (%)	17 (27%)	11 (58%) †	0.01
*Pseudomonas aeruginosa*, number (%)	16 (25%)	0 (0%)	0.02*
MSSA, number (%)	16 (25%)	0 (0%)	0.02*
MRSA, number (%)	9 (14%)	0 (0%)	0.19*
*Haemophilus influenzae*, number (%)	4 (6%)	0 (0%)	0.57*
*Stenotrophomonas maltophilia*, number (%)	13 (20%)	0 (0%)	0.06*
*Achromobacter xylosoxidans*, number (%)	2 (3%)	0 (0%)	0.99*
*Burkholderia cepacia*, number (%)	1 (2%)	0 (0%)	0.99*
*Nontuberculous mycobacteria*, number (% positive with NTM testing) (number with test done)	5 (8%) (N=60)	1 (5%) (N=22)	0.99*
Antibiotic Use number (% of those with data available) (number with data available)	40 (63%) (N=64)	4 (20%) (N=20)	<0.01*

CF, cystic fibrosis; FEV1, forced expiratory volume in 1 s; BALF, bronchoalveolar lavage fluid; MSSA, methicillin-susceptible Staphylococcus aureus; MRSA, methicillin-resistant Staphylococcus aureus; N/A, not applicable. *P-value calculated using Fisher’s exact test †Of the 8 positive cultures: 2 detected Actinomyces; 2 Streptococcus Pneumonia; 2 Mixed Upper Respiratory Flora; 1 Beta Hemolytic Strep Group A and 1 Moraxella catarrhalis & streptococcus pneumoniae.

### Metabolomics Analysis

409 features were measured in BALF. There were 470 (1.3%) values outside the limit of detection. Five acylcarnitines were excluded for having over 50% of the values outside the limit of detection, and an additional 3 features, including 2 glycerophospholipids and 1 sphingolipid, were excluded for having constant values across the sample set, resulting in a total of 401 features being included in the analysis.

**Figure 1:**
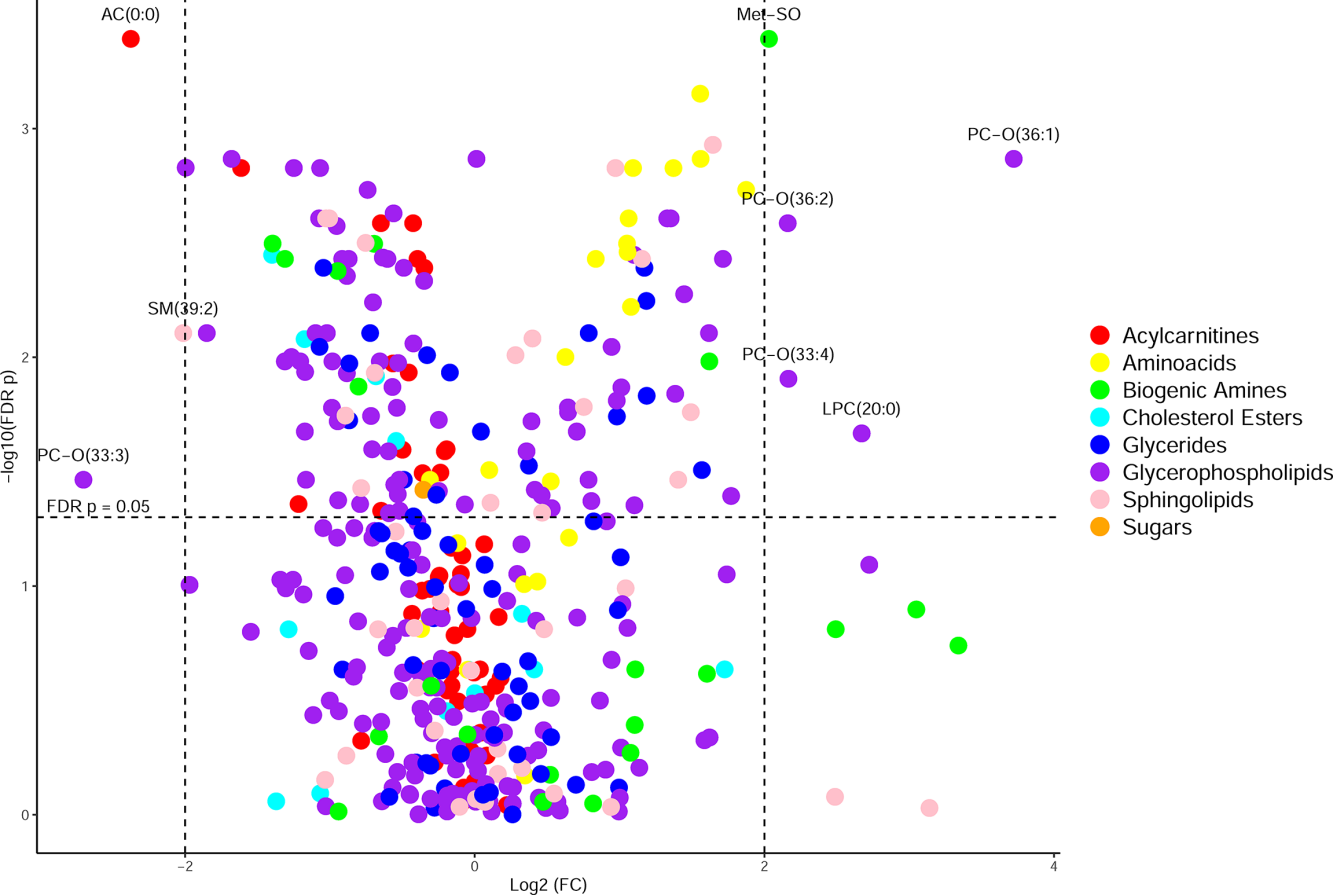
Volcano plot of metabolites organized by class, with the y-axis being -log_10_(FDR p-value) from a Wilcoxon rank sum test and the x-axis being log_2_(fold change) of the values prior to column-wise normalization. Points higher up on the y axis indicate features with greater significance. Points on the left side indicate features found in less abundance in CF samples and points on the right side indicate features found in greater abundance in CF samples.

**Figure 2:**
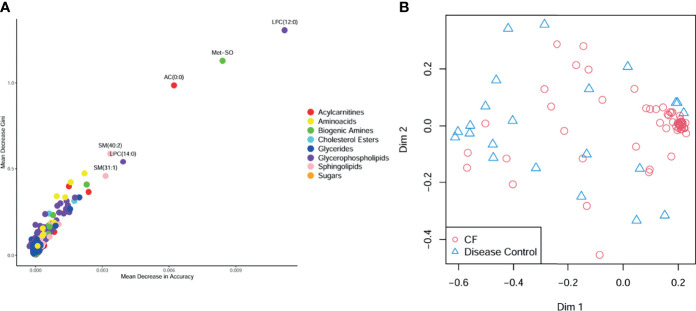
**(A)** Random forest multiway importance plot showing mean decrease Gini verses mean decrease in accuracy, with important metabolic features identified by both criteria labeled and point colored by class **(B)** multidimensional scaling plot of the proximity matrix, red circles corresponding to CF samples, and blue triangles corresponding to disease control samples.

CF BALF had notably higher concentrations of amino acids and lower concentrations of acylcarnitines compared to DCs (**Figure 1**). Additional differences included several glycerophospholipids as well as sphingomyelin (39:2), which were significantly higher in the DC group (FDR p-value = 0.01), and L-methionine S-oxide, which was significantly higher in the CF group (FDR p-value < 0.01). Random forest classification using Biocrates metabolomic features had a predictive accuracy of 81.1% in classifying CF and DC samples. Strong predictors of CF status from the random forest included lysophosphatidylcholine (12:0), the biogenic amine L-methionine S-oxide (Met-SO), and acylcarnitine (0:0) (**Figure 2A**). Lysophosphatidylcholine (12:0) and acylcarnitine (0:0) concentrations were lower and L-methionine S-oxide concentrations were higher in PWCF (**Supplementary Figure 1**). The first two dimensions of the multidimensional scaling (MDS) plot from the proximity matrix indicate a lack of separation between PWCF and DC subjects for about one-third of the CF samples, the remaining CF samples are grouped in a distinct cluster (**Figure 2B**).

### Correlations Between Metabolomic Features and Measures of Infection and Inflammation

**Figure 3:**
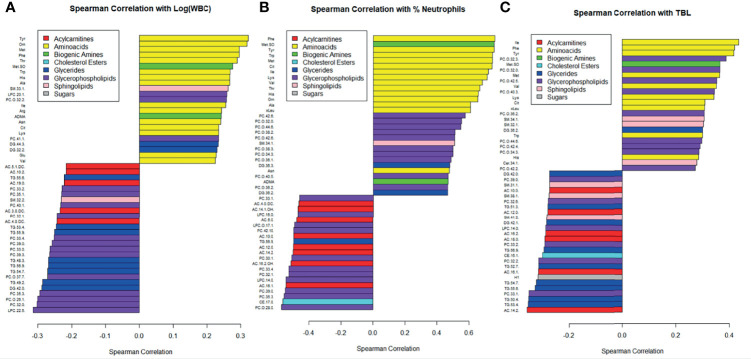
Correlations between metabolite concentrations and **(A)** the logarithm of white blood cell count, **(B)** percent neutrophils, and **(C)** TBL. Bottom of the y-axis are the most negatively correlated and the top of the y-axis are the most positively correlated. Metabolites are color coded by class, and only the 50 most strongly correlated metabolites were included.

The 50 metabolomic features most strongly correlated with white blood cell count and percent neutrophils are displayed in **Figure 3**. Amino acids were among the metabolomic features most positively correlated with white blood cell count and percent neutrophils, while glycerides, glycerophospholipids, and acylcarnitines were among the features most negatively correlated (**Figures 3A, B**). Similar correlations were observed with total bacterial load, as measured by qPCR (**Figure 3C**). The Spearman correlations broken down by CF status are displayed in **Supplementary Figure 2**.

### Sequencing and Network Analysis

**Figure 4:**
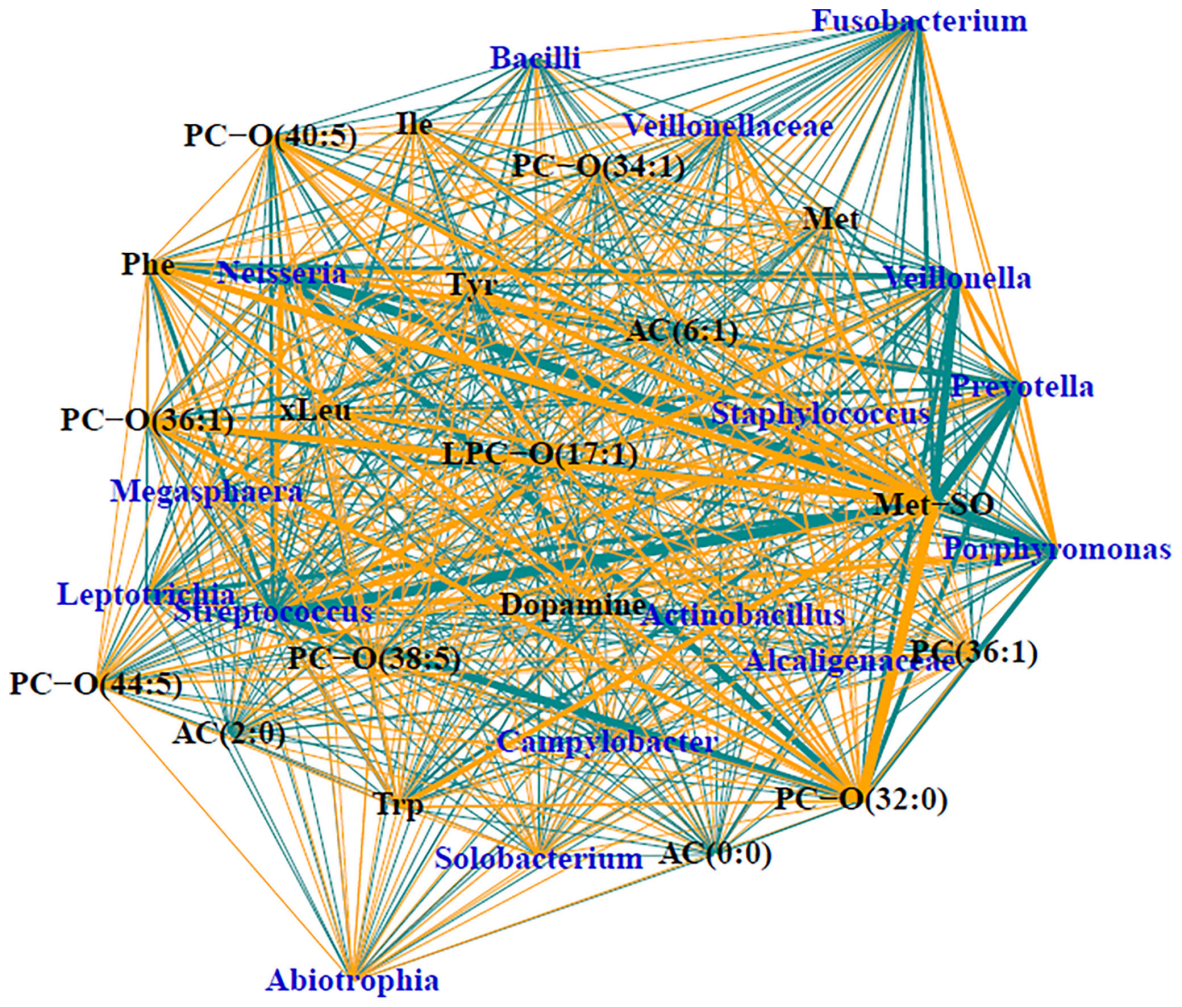
Trimmed module subnetwork identifying microbiome-metabolome correlations with the CF phenotypic outcome. Yellow edges indicate positive correlations and turquoise edges indicate negative correlations. Wider network edges indicate stronger correlations. Blue nodes are taxa identified in 16S and black nodes are metabolites.

Of the 90, 57 of the samples had sufficient load for sequencing, which included 47 (70%) of the CF samples and 10 (45%) of the DC samples. Demographics and relevant clinical characteristics for the samples with sufficient load for sequencing are presented in **Supplementary Table 2**. Relative abundances are displayed in **Supplementary Figure 3**. The median number of taxa identified in the entire sample set was 18, with the number of taxa identified ranging from 2 to 82. When split by CF status, medians (ranges) of taxa detected included 17 (2-74) taxa for CF samples and 55 (10-82) taxa for DC samples (p<0.01). Sparse supervised canonical correlation analysis (SsCCA) performed on the entire sample set revealed subnetwork variation between PWCF and DCs. Notably, the networks contain mostly weak associations between metabolomic features and microbial taxa. The subnetwork with the strongest correlations is shown in **Figure 4**, and included a subnetwork with 16 taxa nodes, including the traditional CF pathogen, *Staphylococcus*, and nontraditional pathogens, *Prevotella*, *Streptococcus*, and *Veillonella*, that were correlated with a network of 19 metabolomic features, including 8 glycerophospholipids, 6 amino acids, 2 biogenic amines, and 3 acylcarnitines. Notably, in PWCF, L-methionine S-oxide was negatively correlated with the anaerobic taxa *Prevotella*, *Streptococcus*, and *Veillonella* and positively correlated with the traditional CF-pathogen *Staphylococcus* (**Supplementary Figure 4**). An additional subnetwork is included in the supplementary material (**Supplementary Figure 5**).

**Figure 5:**
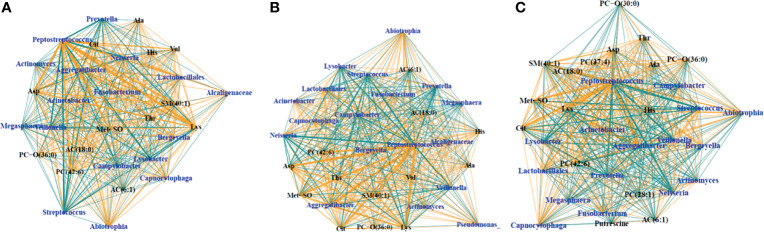
Trimmed module subnetworks identifying microbiome metabolome correlations with no phenotypic outcome **(A)**, WBC phenotype **(B)** and percent neutrophil outcome **(C)**. Yellow edges indicate positive correlations and turquoise edges indicate negative correlations. Wider network edges indicate stronger correlations. Blue nodes are taxa identified in 16S and black nodes are metabolites.

SsCCA was performed again on just the samples from PWCF, using no phenotypic outcome as well as white blood cells and percent neutrophils as the phenotypic outcomes (**Figure 5**). Interestingly, similar networks were identified in the CF samples, regardless of phenotype. Each analysis resulted in one subnetwork with taxa nodes including nontraditional CF pathogens, *Fusobacterium*, *Neisseria*, *Veillonella*, *Prevotella*, and *Streptococcus* that were correlated with a mixed network of amino acids, glycerophospholipids, acylcarnitines, and sphingolipids (**Figure 5**). Additional subnetworks are included in the supplementary material (**Supplementary 6**).

## Discussion

In this study, we harnessed the power of metabolomics and microbiome data to comprehensively assess the complex interplay between infection and inflammation in the lower airways of PWCF across the age spectrum. We determined differences in the lower airway metabolome between PWCF and DC subjects using the gold standard BALF sample. By characterizing the metabolome in BALF with the Biocrates platform, we identified metabolomic characteristics unique to the lower CF airway and identified relationships between metabolomic features and measures of infection and inflammation. By integrating metabolomics data with microbiome data acquired through 16S sequencing, we were able to determine hypothesis-generating multi-omics correlations. Based on previous work, we expected to see correlations between the microbiome and metabolomic markers associated with inflammation, proteolytic activity, and anaerobic glycolysis as has been previously shown in sputum (Twomey et al., 2013; Zemanick et al., 2015; Quinn et al., 2019). In our study, using BALF, we expanded upon previous findings and identified additional complex relationships between metabolomic features and bacterial communities present in the lower airways of PWCF and DC subjects providing unique insight into the active metabolism within the airway environment.

The metabolome of the CF airway had increased amino acids and decreased acylcarnitines compared to DC children, identifying potential biomarkers of an inflamed CF lower airway. Previous studies have shown increased amino acid concentrations in CF serum and sputum (Barth and Pitt, 1996; Masood et al., 2021), as well as decreased acylcarnitine concentrations in CF plasma (Kovesi et al., 1994). Our findings demonstrate the potential utility of using acylcarnitine and amino acid concentrations as biomarkers of CF, indicating there may be key differences in amino acid and fatty acid metabolism in the CF airway. Amino acids and acylcarnitines were also correlated with inflammation, as measured by white blood cell count and percent neutrophils, and bacterial burden, as measured by qPCR, in the entire sample set and specifically in CF BALF samples. The correlation between inflammation and amino acid concentration has been well documented in CF (Wolak et al., 2009; Esther et al., 2015) and amino acids have been known to play a crucial role in host-pathogen metabolomic crosstalk (Ren et al., 2018). Acylcarnitines have also been implicated in inflammatory signaling (Rutkowsky et al., 2014), suggesting that those two defining features of the lower CF airway may coincide with altered CF immune response to infection. Random forest analyses revealed strong predictors of a CF status in the airway, including lysophosphatidylcholine (12:0), which was found to be in lower concentrations in PWCF. Decreased lysophosphatidylcholine has been previously reported in CF tracheobronchial secretions (Slomiany et al., 1982), so our results further emphasize altered lipid metabolism in the CF airway (Yang et al., 2012). Another predictor of CF status, L-methionine-S-oxide, is a byproduct of the oxidation of methionine by myeloperoxidase in periods of oxidative stress (Pattison and Davies, 2001; Denkel et al., 2011) and is correlated with structural lung damage and airway neutrophils in early stages of CF (Chandler et al., 2018). Overall, our characterization of the CF metabolome paints a picture of a highly inflamed lower airway where enhanced immune response and corresponding neutrophilic influx may be resulting in more free amino acids, altered lipid metabolism, and increased production of damaging reactive oxygen species.

SsCCA revealed complex and intriguing multi-omic correlations between the metabolome and the microbiome, characterized by 16S sequencing. In the trimmed subnetwork with the strongest correlations, we observed relationships between *Staphylococcus*, a known CF pathogen, anaerobic taxa, including *Prevotella*, *Streptococcus*, *Veillonella*, and *Fusobacterium*, and a subnetwork of metabolomic features made up of mostly amino acids and glycerophospholipids. Previous findings have already shown traditional CF pathogens to be associated free peptides and amino acids in sputum (Quinn et al., 2019), however our work expanded on that identifying anaerobic bacteria as other correlates with airway proteolytic activity. Among the other features in the subnetwork were acylcarnitine (0:0) and L-methionine-S-oxide, two of the strongest predictors of a CF status in BALF from random forest, which suggests that the composition of the CF airway metabolome may be defined by the polymicrobial bacterial communities present within it.

L-methionine-S-oxide displayed the strongest correlations in the subnetwork of all BALF samples, being strongly positively correlated with *Staphylococcus* and negatively correlated with anaerobes in the CF lower airway. Review of the scatter plots of the L-methionine-S-oxide indicated that, while strong correlations were observed in the normalized data, unnormalized data had varying ranges of the metabolite present. L-methionine-S-oxide was decreased in the DC samples with CF subjects having an increased range of concentrations measured. In CF BALF, decreased concentrations of this metabolite were associated with varying relative abundances of *Prevotella*, *Veillonella*, and *Streptococcus*, while increased concentrations were associated with significantly decreased abundances of those taxa. Therefore, the concentration of this L-methionine-S-oxide biomarker appears to be dependent on other taxa present, like *Staphylococcus*, which was positively correlated in CF, confirming the likely polymicrobial nature of the lower CF airway. The additional positive correlations observed between L-methionine-S-oxide and other amino acids in the subnetwork further demonstrates the potential utility of using that biogenic amine as a biomarker of infection and inflammation in CF lung disease, which has been previously shown (Chandler et al., 2018). Previous studies have demonstrated an inverse relationship between the presence of anaerobes and traditional CF pathogens (Zemanick et al., 2017; O’Connor et al., 2021), with *in vitro* studies suggesting anaerobes prime the CF lung for chronic infection of traditional pathogens like *Pseudomonas aeruginosa* (Flynn et al., 2016). Therefore, the dual opposite correlations observed between L-methionine-S-oxide and anaerobes and *Staphylococcus*, along with its correlation with infection and inflammation, indicate that L-methionine S-oxide could be a crucial metabolite involved in microbial cross talk. It could be a defining feature of the CF airway metabolome that correlates with the CF lung’s intense inflammation and enhanced bacterial burden which sets the stage for chronic infection and lung damage and should be further investigated.

Additional multi-omic networks in this study demonstrated several weaker relationships between metabolomic features and taxa, including the subnetwork demonstrating correlations between traditional CF pathogens *Pseudomonas* and *Stenotrophomonas* and amino acids within the CF BALF samples, which merits further investigation. When observing subnetworks found in only the CF samples using unsupervised SsCCNet and SsCCNet incorporating inflammation as the phenotypic variable, we saw similar classes of weaker subnetworks with and without the inflammation phenotype. This suggests that even when inflammation is not specifically incorporated into the correlation analysis, the same subnetworks of typical CF pathogens are seen, indicating that inflammation may be a natural driver of metabolome-microbiome correlations.

Our study is not without limitations. BALF samples were collected from multiple sites, so although processing was conducted in the same way, sample collection was not performed uniformly at each institution. Similarly, because all BALF samples were collected from clinically indicated bronchoscopies mostly during periods of illness, metabolomic characterization of these samples is not representative of baseline stability. PWCF were also more frequently undergoing antibiotic treatment at the time of sample collection, which can impact the features of the airway metabolome (Hahn et al., 2020; Raghuvanshi et al., 2020). Furthermore, PWCF were slightly older in age at collection, which can correspond to changes in the airway microbiome (Zemanick et al., 2017; O’Connor et al., 2021). While changes in the airway metabolome have been observed in short longitudinal studies (Hahn et al., 2020; Raghuvanshi et al., 2020), the dynamics of the CF airway metabolome across the age spectrum require further investigation. Additionally, because our DC subjects, which were limited in sample number, had a variety of diagnoses and clinical indications, our findings in that cohort are not representative of a healthy lower airway metabolome and are limited by the heterogeneity of the underlying diseases and sample size. Lastly, we used the CCA analyses to uncover potential correlations between the metabolome and the microbiome, which required that the data be transformed. Therefore, our results may be harder to interpret. We presented the associations on the raw scale in an attempt to address this limitation. We recognized alternative approaches to multi-omics analysis, but we chose the CCA approach because it allowed us to estimate correlations between omics and to consider a phenotype simultaneously.

In this hypothesis-generating study, by performing targeted metabolomics of BALF samples obtained from PWCF and DC subjects across the age spectrum, we provided unique insight into the lower CF airway metabolome. While our analysis prevents us from drawing strong inferences, our observations suggest the CF airway’s hyperimmune response and corresponding neutrophilic inflammation may be resulting in more free amino acids, changes in lipid metabolism, and the production of reactive oxygen species damaging to the lower airway. By integrating BALF metabolomic data and 16S sequencing data as well as phenotypic outcome measures, we were able to use a modern multi-omics approach to support the likely polymicrobial nature of the CF lower airway and elucidate the complex relationships between the microbiome, the metabolome, and CF inflammation.

## Data Availability Statement

The datasets presented in this study can be found in online repositories. The names of the repository/repositories and accession number(s) can be found below: https://www.ncbi.nlm.nih.gov/, PRJNA638906.

## Author Contributions

TA, JH, CW, and BW contributed to conception and design of the study. AM performed the sample preparation. MK performed the Biocrates analysis. JH performed the 16S analysis and the qPCR. JO’C managed the sample database and sample transfers between institutions. JO’C and MM organized the database. BW and JO’C performed the statistical analysis. JO’C wrote the first draft of the manuscript. BW, JH, and MK wrote sections of the manuscript. All authors contributed to manuscript revision, read, and approved the submitted version.

## Funding

This work was supported by grants from the National Institutes of Health (NIH R01HL136499) and the Cystic Fibrosis Foundation (CFF LAGUNA17A0). The funders had no role in the design of the study, data collection and analysis, publication decisions, or preparation of the manuscript.

## Author Disclaimer

The views expressed in this article are those of the authors and do not reflect the views of the United States Government, the Department of Veterans Affairs, the funders, the sponsors, or any of the authors’ affiliated academic institutions.

## Conflict of Interest

The authors declare that the research was conducted in the absence of any commercial or financial relationships that could be construed as a potential conflict of interest.

## Publisher’s Note

All claims expressed in this article are solely those of the authors and do not necessarily represent those of their affiliated organizations, or those of the publisher, the editors and the reviewers. Any product that may be evaluated in this article, or claim that may be made by its manufacturer, is not guaranteed or endorsed by the publisher.
