# Supplementary figures and images for: Network Analysis to Identify Multi-Omic Correlations in the Lower Airways of Children With Cystic Fibrosis
Source: Front Cell Infect Microbiol. 2022 Mar 10;12:805170. doi: 10.3389/fcimb.2022.805170 (PMC8960254; doi:10.3389/fcimb.2022.805170)

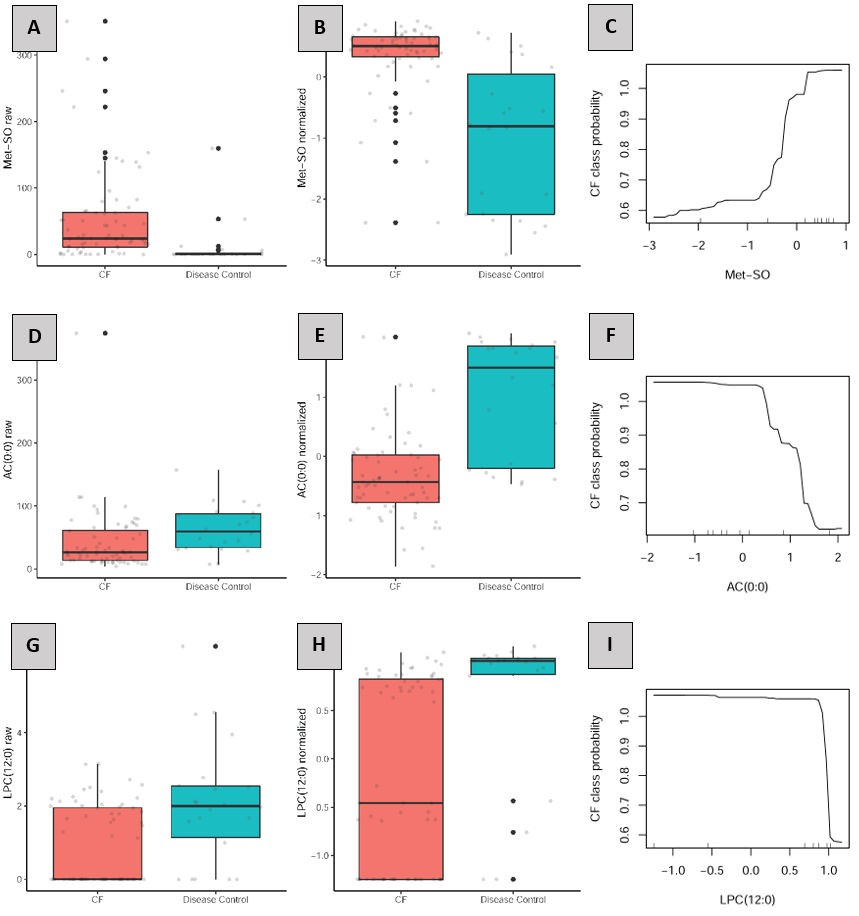

Supplement: Supplementary file 1 [file Image_1.tiff]

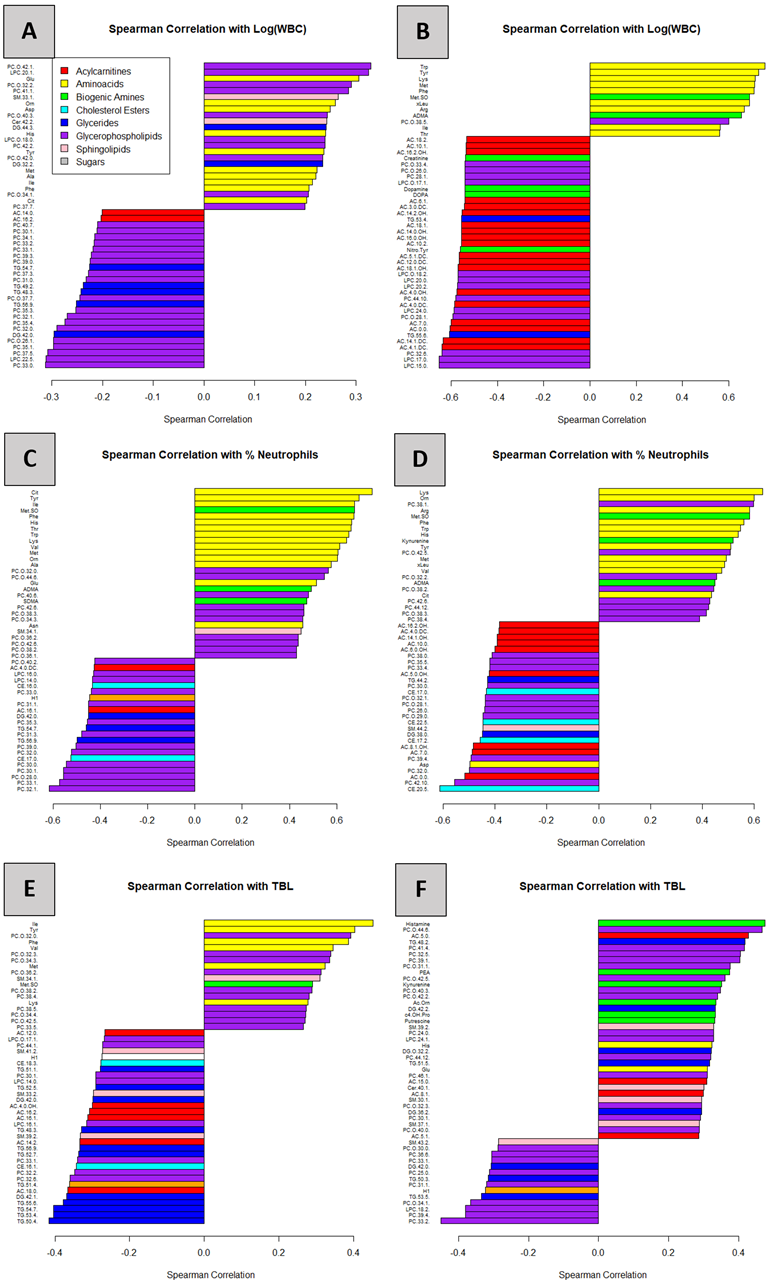

Supplement: Supplementary file 2 [file Image_2.tiff]

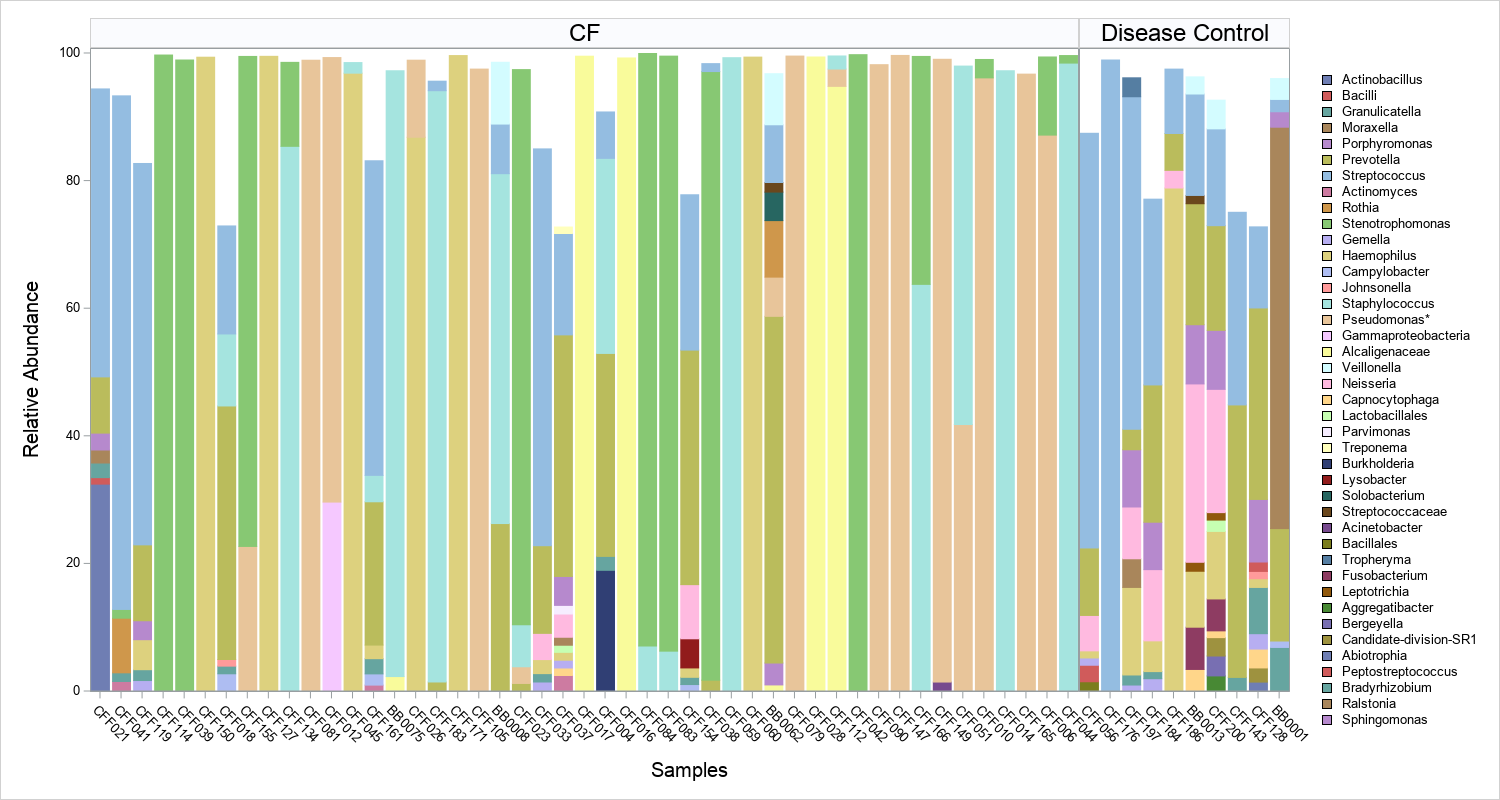

Supplement: Supplementary file 3 [file Image_3.tif]

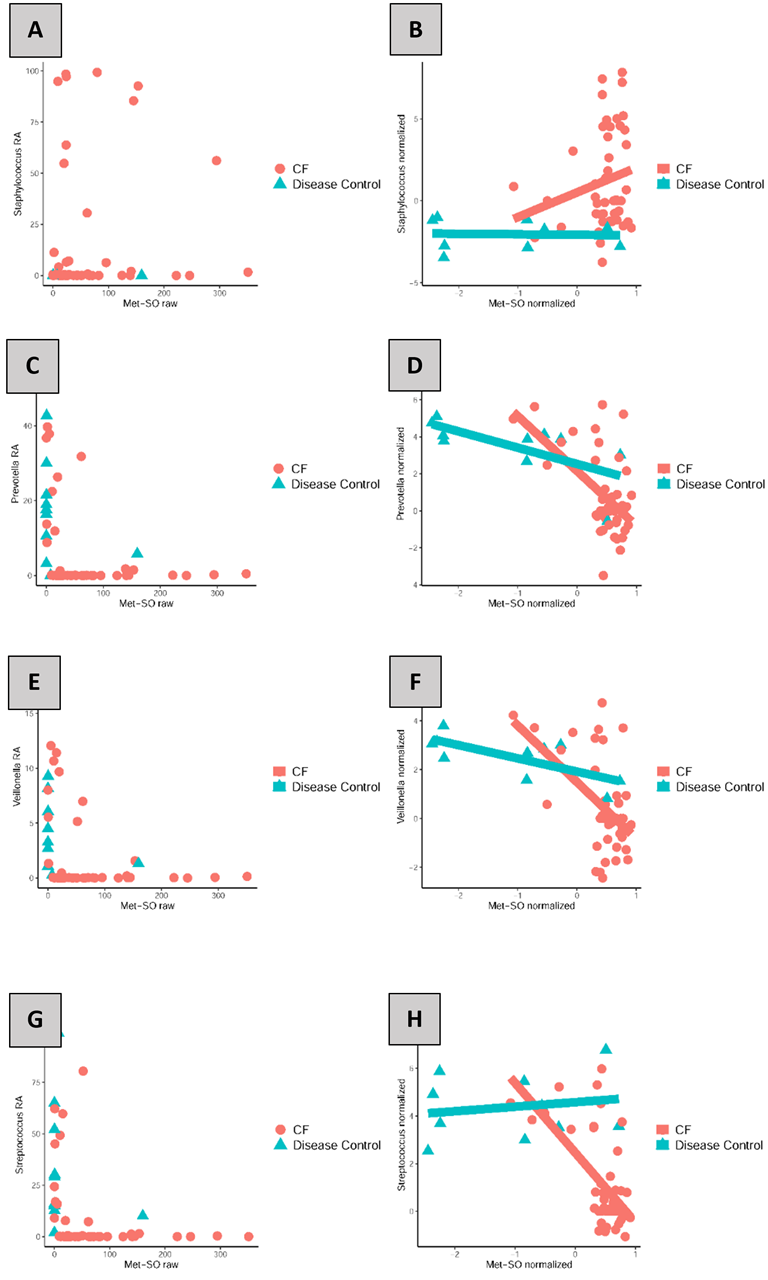

Supplement: Supplementary file 4 [file Image_4.tif]

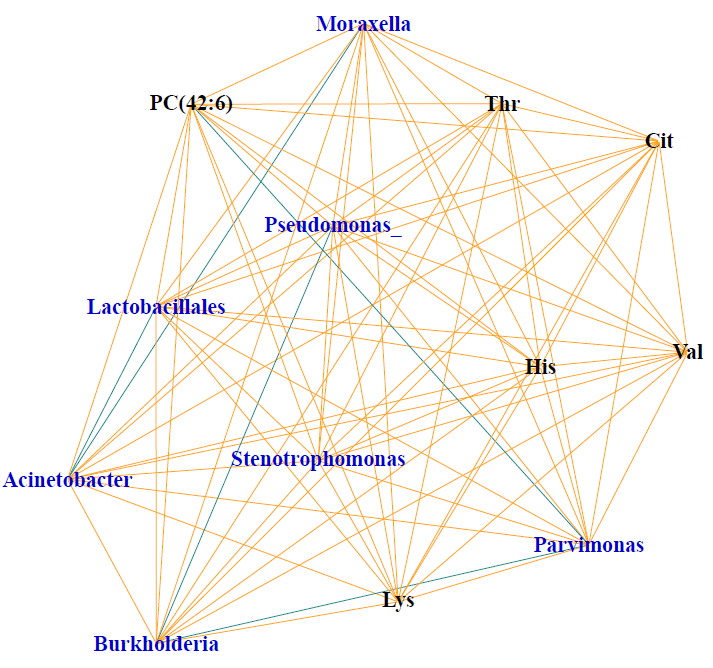

Supplement: Supplementary file 5 [file Image_5.tif]

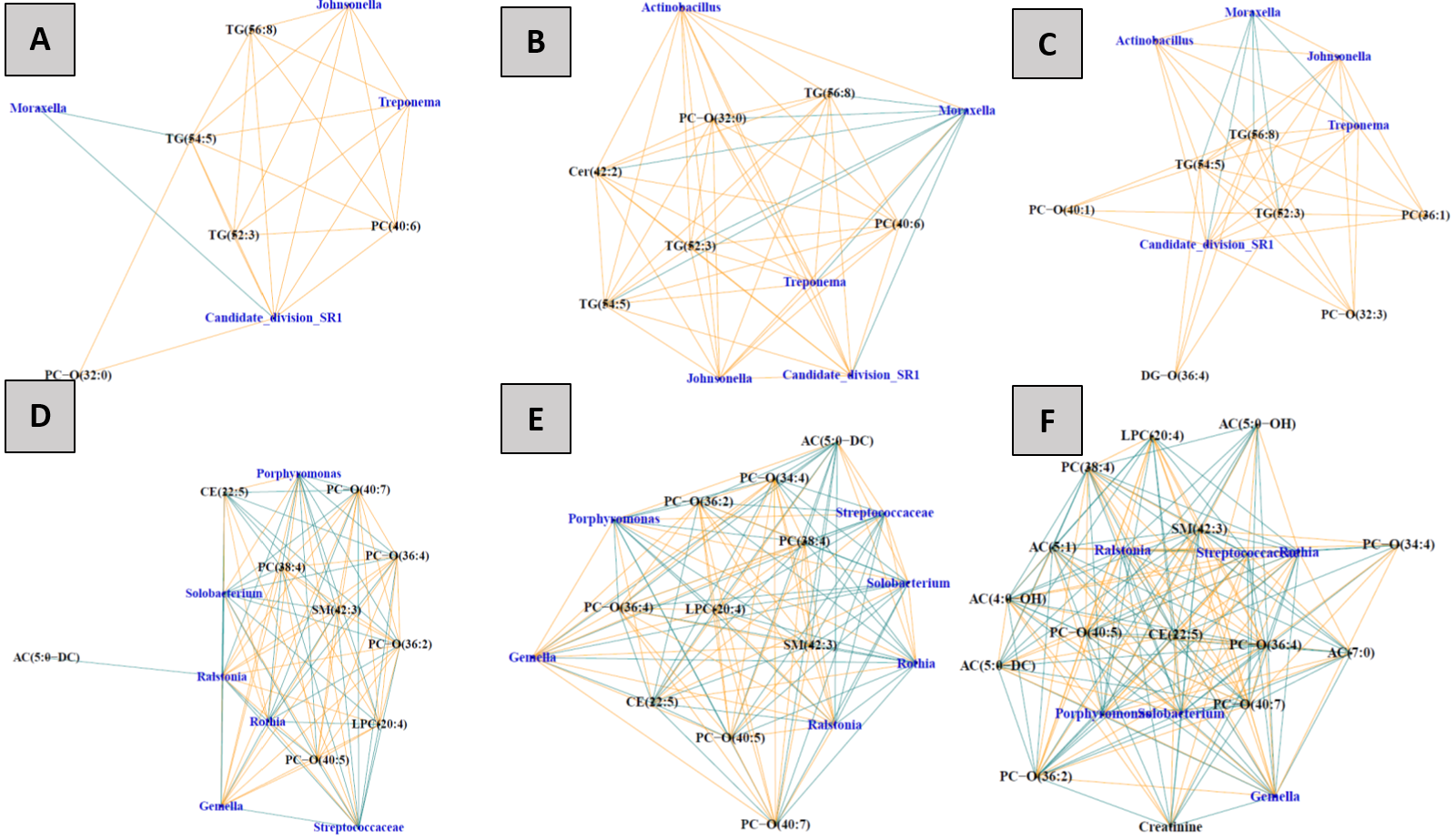

Supplement: Supplementary file 6 [file Image_6.tiff]
