# Supplementary material for: Network Analysis to Identify Multi-Omic Correlations in the Lower Airways of Children With Cystic Fibrosis
Source: Front Cell Infect Microbiol. 2022 Mar 10;12:805170. doi: 10.3389/fcimb.2022.805170 (PMC8960254; doi:10.3389/fcimb.2022.805170)
Supplement: Supplementary file 7 [file DataSheet_1.docx]

Supplementary Material

# Supplementary Methods

## DNA Extraction and Quantitative Polymerase Chain Reaction (qPCR)

The Qiagen EZ1 Advanced automated extraction platform (Qiagen, Valencia, CA, USA) was used to extract DNA from the samples in accordance with the manufacturer’s instructions. Quantitative Polymerase Chain Reaction (qPCR) was used to assess total bacterial load (TBL), which has been previously reported and validated in CF samples (Nadkarni et al., 2002; Zemanick et al., 2010, O’Connor et al., 2021). To account for the presence of human DNA in airway samples and maximize measurement of microbial DNA, TBL was used to assess template requirements for amplification of the 16S rRNA gene. Samples with extracted DNA that was less than 1.5 times the plate background were considered quantity not sufficient and were excluded from amplicon generation.

## High-throughput Sequencing for Microbiota Analysis

Broad range amplification and sequence analysis of 16S rRNA was used to profile airway bacterial taxa in a process previously reported (Hara et al., 2012; Markle et al., 2013; Laguna et al., 2016; Zemanick et al., 2017). To assess background, negative control samples were included in sequencing. MiSeq compatible primers targeting approximately 300 base pairs in the V1/V2 variable region (27F to 338R) of the 16S rRNA gene were used to generate amplicons. Agarose gel densitometry was used to normalize PCR products, which were then pooled, gel purified and concentrated using a DNA Clean and Concentrator Kit (Zymo, Irvine, CA). The Qubit Fluorometer 2.0 (Invitrogen, Carlsbad, CA) was used to quantify the pooled amplicons, which were then diluted to 4 nM and denatured at room temperature with 0.2 N NaOH. Before loading it on the sequencer, the denatured DNA was further diluted to 20 pM and spiked with 10% of the Illumina PhiX control DNA. The MiSeq platform was used to perform Illumina paired-read sequencing with a 500-cycle version 2 reagent kit.

## Analysis of Illumina Paired end Reads

Bowtie2 was used to align paired end reads to the human reference genome Hg19 and matching sequenced were discarded (Website; Langmead and Salzberg, 2012). The remaining paired-end sequences that were not matched to the human genome were sorted via sample barcodes in the paired-reads as has been previously described (Markle et al., 2013). After paired-end sequences were sorted, data was entered into the NCBI Short Read Archive (Accession number- PRJNA638906). Phrap was used to assemble paired-end sequences (Ewing and Green, 1998; Ewing et al., 1998), with unassembled sequences being discarded. Trimming was performed over a moving window of 5 nucleotides until a quality above or equal to 20 was achieved, and any trimmed sequences shorter than 200 nt or having more than 1 ambiguity were excluded. Using the Schloss (Schloss and Westcott, 2011) Silva reference sequence with Uchime (usearch6.0.203_i86linux32) (Edgar et al., 2011), potential chimeras were identified and removed from analysis. Silva taxonomy was yielded by aligning unique assembled sequences with 418,497 reference bacterial sequences in Silva 115NR99 (Quast et al., 2013) and classifying them with SINA (1.3.0-r23838) (Pruesse et al., 2012). Grouping sequences with identical taxonomic assignments yielded operational taxonomic units.

# Supplementary Results

Demographics and relevant clinical characteristics for the samples with sufficient load for sequencing are presented in **Supplementary Table 2**. These samples represent a subset of samples in which PWCF were older (p=0.04) than DCs with increased white blood cell count (p<0.01).

SsCCNet of all samples incorporating the CF phenotype resulted in additional subnetworks identified. It resulted in one subnetwork with 7 taxa nodes, including traditional CF pathogens like *Burkholderia*, *Pseudomonas*, and *Stenotrophomonas*, that were correlated with a network of 6 metabolomic features, that included mostly amino acids (**Supplementary Figure 5**).

SsCCNet on only the CF samples both unsupervised (no phenotypic outcome) and incorporating inflammation as the phenotypic variable are shown in **Supplementary Figure 6**. The three analyses each resulted in another subnetwork with taxa nodes including *Actinobacillus*, *Moraxella* and *Treponema* that were correlated with a network of glycerides and glycerophospholipids (**Supplementary Figure 6 A-C**) as well as a subnetwork of taxa nodes including *Ralstonia*, *Porphyromonas*, and *Gemella* that were correlated with a network of acylcarnitines, sphingolipids, glycerophospholipids, and the cholesterol ester CE(22:5) (**Supplementary Figure 6 D-F**).

# References

Edgar, R. C., Haas, B. J., Clemente, J. C., Quince, C., and Knight, R. (2011). UCHIME improves sensitivity

and speed of chimera detection. Bioinformatics 27, 2194–2200.

Ewing, B., and Green, P. (1998). Base-calling of automated sequencer traces using pared. II. Error

probabilities. Genome Res. 8, 186–194.

Ewing, B., Hillier, L., Wendl, M. C., and Green, P. (1998). Base-calling of automated sequencer traces

using phred. I. Accuracy assessment. Genome Res. 8, 175–185.

Hara, N., Alkanani, A. K., Ir, D., Robertson, C. E., Wagner, B. D., Frank, D. N., et al. (2012). Prevention of

virus-induced type 1 diabetes with antibiotic therapy. J. Immunol. 189, 3805–3814.

Laguna, T. A., Wagner, B. D., Williams, C. B., Stevens, M. J., Robertson, C. E., Welchlin, C. W., et al.

(2016). Airway Microbiota in Bronchoalveolar Lavage Fluid from Clinically Well Infants with Cystic Fibrosis. PLoS One 11, e0167649.

Langmead, B., and Salzberg, S. L. (2012). Fast gapped-read alignment with Bowtie 2. Nat. Methods 9, 357–359.

Markle, J. G. M., Frank, D. N., Mortin-Toth, S., Robertson, C. E., Feazel, L. M., Rolle-Kampczyk, U., et al.

(2013). Sex differences in the gut microbiome drive hormone-dependent regulation of autoimmunity. Science 339, 1084–1088.

Nadkarni, M. A., Martin, F. E., Jacques, N. A., and Hunter, N. (2002). Determination of bacterial load by

real-time PCR using a broad-range (universal) probe and primers set. Microbiology 148, 257–266.

Pruesse, E., Peplies, J., and Glöckner, F. O. (2012). SINA: accurate high-throughput multiple sequence

alignment of ribosomal RNA genes. Bioinformatics 28, 1823–1829.

Quast, C., Pruesse, E., Yilmaz, P., Gerken, J., Schweer, T., Yarza, P., et al. (2013). The SILVA ribosomal RNA

gene database project: improved data processing and web-based tools. Nucleic Acids Res. 41, D590–6.

Schloss, P. D., and Westcott, S. L. (2011). Assessing and improving methods used in operational

taxonomic unit-based approaches for 16S rRNA gene sequence analysis. Appl. Environ. Microbiol. 77, 3219–3226.

Website Available at: 55. iGenomes. At

<https://support.illumina.com/sequencing/sequencing_software/igenome.html>.

Zemanick, E. T., Wagner, B. D., Robertson, C. E., Ahrens, R. C., Chmiel, J. F., Clancy, J. P., et al. (2017).Airway microbiota across age and disease spectrum in cystic fibrosis. Eur. Respir. J. 50. doi:10.1183/13993003.00832-2017.

Zemanick, E. T., Wagner, B. D., Sagel, S. D., Stevens, M. J., Accurso, F. J., and Harris, J. K. (2010).

Reliability of quantitative real-time PCR for bacterial detection in cystic fibrosis airway specimens. PLoS One 5, e15101.

# Tables

**Supplementary Table 1:** Indications and Primary diagnoses of DC subjects sorted by frequency. Indication/primary diagnosis categories were not mutually exclusive with subjects falling into as many as 3 different indications.

| **Indication** | **N (%)** |
| --- | --- |
| Airway evaluation | 11 (50%) |
| Unknown/other | 6 (27%) |
| Rule-out infection | 4 (18%) |
| Oncology/Immunosuppressed/immunodeficiency | 4 (18%) |
| Cough | 2 (9%) |
| Hemoptysis | 2 (9%) |
| Asthma | 1 (5%) |

**Supplementary Table 2:** Data are presented as n, median (range) or n (%), unless otherwise stated. CF: cystic fibrosis; FEV1: forced expiratory volume in 1 s; BALF: bronchoalveolar lavage fluid; MSSA: methicillin-susceptible *Staphylococcus aureus*; MRSA: methicillin-resistant *Staphylococcus aureus*. *P-value calculated using Fisher’s exact test

|  | CF (n = 47) | Disease Control (n = 10) | P-value |
| --- | --- | --- | --- |
| Age years, median (range) | 13.0 (1.0-28.0) | 7.2 (1.3-17.8) | 0.04 |
| <2 years, number (%) | 2 (4%) | 2 (20%) | 0.06* |
| 2-5 years, number (%) | 4 (9%) | 1 (10%) |  |
| 6-10 years, number (%) | 11 (23%) | 5 (50%) |  |
| 11-17 years, number (%) | 19 (40%) | 2 (20%) |  |
| 18 years and older, number (%) | 11 (23%) | 0 (0%) |  |
| Female, number (%) | 20 (43%) | 5 (50%) | 0.67 |
| Weight (kg), median (range) (data available) | 46.8 (12.3-87.0) (N=44) | 20.4 (9.9-62.1) (N=10) | 0.02 |
| Height (cm), median (range) (data available) | 155.6 (79.0-185.4) (N=44) | 118.2 (74.0-170.9) (N=10) | 0.05 |
| Genotype, data available | N=40 | N/A | ___ |
| F508del/F508del, number (%) | 22 (55%) | N/A | ___ |
| F508del/other, number (%) | 13 (33%) | N/A | ___ |
| Other/other, number (%) | 5 (13%) | N/A | ___ |
| FEV1 % predicted, median (range) (data available) | 83.0 (44.0-125.0) (N=37) | 80.0 (38.0-121.0) (N=5) | 0.85 |
| BALF Cell Counts, data available | N=68 | N=22 |  |
| White blood cells, median (range) (data available) | 1364.5 (68.0-41167.0) (N=34) | 214.5 (52.0-1125.0) (N=10) | <0.01 |
| Percent Neutrophils, median (range) (data available) | 75.0 (10.0-98.0) (N=39) | 6.0 (1.0-100.0) (N=10) | <0.01 |
| Percent Lymphocytes, median (range) (data available) | 2.0 (0-28.0) (N=37) | 11.5 (0-37.0) (N=10) | 0.04 |
| BALF culture results, data available | N=44 | N=10 |  |
| Negative, number (%) | 5 (11%) | 4 (40%) | 0.05* |
| Pseudomonas aeruginosa, number (%) | 16 (36%) | 0 (0%) | 0.05* |
| MSSA, number (%) | 13 (30%) | 0 (0%) | 0.18* |
| MRSA, number (%) | 6 (14%) | 0 (0%) | 0.57* |
| Haemophilus influenzae, number (%) | 3 (7%) | 0 (0%) | 1.00* |
| Stenotrophomonas maltophilia, number (%) | 12 (27%) | 0 (0%) | 0.17* |
| Achromobacter xylosoxidans, number (%) | 2 (5%) | 0 (0%) | 1.00* |
| Burkholderia cepacia, number (%) | 1 (2%) | 0 (0%) | 1.00* |
| Nontuberculous mycobacteria, number (% positive with NTM testing) (number with test done) | 4 (10%) (N=42) | 0 (0%) (N=10) | 0.58* |
| Antibiotic Use number (% of those with data available) (number with data available) | 27 (61%) (N=44) | 1 (10%) (N=10) | <0.01* |

# Figure Legends

**Supplementary Figure 1:** Box plots of raw values (A,D,G) and normalized values (B,E,H) across disease groups and partial plots from the random forest (C,F,I) illustrating the association between normalized metabolite values and the CF class predictions.

**Supplementary Figure 2:** Correlations between metabolite concentrations and (A,B) the logarithm of white blood cell count (C,D) percent neutrophils, and (E,F) TBL in CF samples (A,C,E) and DC samples (B,D,F). Bottom of the y-axis are the most negatively correlated and the top of the y-axis are the most positively correlated. Metabolites are color coded by class, and only the 50 most strongly correlated metabolites were included.

**Supplementary Figure 3:** Relative abundance of taxa identified in CF and disease control sputum as measured through 16S sequencing.

**Supplementary Figure 4:** Scatter plots with linear trendlines showing the relationship between L-Methionine S-oxide concentration and the relative abundance of *Staphylococcus* (A-B), *Prevotella* (C-D), *Veillonella* (E-F), and *Streptococcus* (G-H). Raw data is displayed on the left (A,C,E,G) and normalized data is displayed on the right (B,D,F,H). CF samples are indicated in red and disease control samples are indicated in blue.

**Supplementary Figure 5:** Additional trimmed module subnetwork identifying microbiome metabolome correlations with the CF phenotypic outcome including CF pathogens of interest. Yellow edges indicate positive correlations and turquoise edges indicate negative correlations. Wider network edges indicate stronger correlation. Blue nodes are taxa identified in 16S and black nodes are metabolites.

**Supplemental Figure 6:** Trimmed module subnetworks identifying microbiome metabolome correlations with no phenotypic outcome (A, D), WBC phenotype (B, E) and percent neutrophil outcome (C, F). Yellow edges indicate positive correlations and turquoise edges indicate negative correlations. Wider network edges indicate stronger correlation. Blue nodes are taxa identified in 16S and black nodes are metabolites.
